# Evaluation of a real-time magnetic resonance imaging-guided electrophysiology system for structural and electrophysiological ventricular tachycardia substrate assessment

**DOI:** 10.1093/europace/euz165

**Published:** 2019-06-20

**Authors:** Rahul K Mukherjee, Caroline Mendonca Costa, Radhouene Neji, James L Harrison, Iain Sim, Steven E Williams, John Whitaker, Henry Chubb, Louisa O’Neill, Rainer Schneider, Tom Lloyd, Thomas Pohl, Sébastien Roujol, Steven A Niederer, Reza Razavi, Mark D O’Neill

**Affiliations:** 1 School of Biomedical Engineering and Imaging Sciences, King’s College London, 4th Floor, North Wing, St Thomas’ Hospital, London, UK; 2 Siemens Healthcare, Sir William Siemens Square, Frimley, Camberley, UK; 3 Department of Cardiology, King’s College Hospital NHS Foundation Trust, London, UK; 4 Department of Cardiology, Guy’s and St Thomas’ NHS Foundation Trust, London, UK; 5 Siemens Healthcare GmbH, Erlangen, Germany; 6 Imricor Medical Systems, 400 Gateway Blvd, MN, USA

**Keywords:** Real time, Magnetic resonance imaging, Electroanatomic mapping, Substrate, Ventricular tachycardia, Late gadolinium enhancement

## Abstract

**Aims:**

Potential advantages of real-time magnetic resonance imaging (MRI)-guided electrophysiology (MR-EP) include contemporaneous three-dimensional substrate assessment at the time of intervention, improved procedural guidance, and ablation lesion assessment. We evaluated a novel real-time MR-EP system to perform endocardial voltage mapping and assessment of delayed conduction in a porcine ischaemia–reperfusion model.

**Methods and results:**

Sites of low voltage and slow conduction identified using the system were registered and compared to regions of late gadolinium enhancement (LGE) on MRI. The Sorensen–Dice similarity coefficient (DSC) between LGE scar maps and voltage maps was computed on a nodal basis. A total of 445 electrograms were recorded in sinus rhythm (range: 30–186) using the MR-EP system including 138 electrograms from LGE regions. Pacing captured at 103 sites; 47 (45.6%) sites had a stimulus-to-QRS (S-QRS) delay of ≥40 ms. Using conventional (0.5**–**1.5 mV) bipolar voltage thresholds, the sensitivity and specificity of voltage mapping using the MR-EP system to identify MR-derived LGE was 57% and 96%, respectively. Voltage mapping had a better predictive ability in detecting LGE compared to S-QRS measurements using this system (area under curve: 0.907 vs. 0.840). Using an electrical threshold of 1.5 mV to define abnormal myocardium, the total DSC, scar DSC, and normal myocardium DSC between voltage maps and LGE scar maps was 79.0 ± 6.0%, 35.0 ± 10.1%, and 90.4 ± 8.6%, respectively.

**Conclusion:**

Low-voltage zones and regions of delayed conduction determined using a real-time MR-EP system are moderately associated with LGE areas identified on MRI.

**What’s new?**

- Endocardial voltage mapping and limited assessments of slow conduction were feasible in a porcine ischaemia–reperfusion model using a novel real-time magnetic resonance imaging-guided electrophysiology system (MR-EP).
- Using conventional bipolar voltage thresholds, there was moderate sensitivity in the ability of voltage mapping with the MR-EP system to identify regions of late gadolinium enhancement (LGE).
- An improved sensitivity for LGE detection may be achieved using higher normal bipolar voltage cut-offs with the MR-EP system and respective MR-compatible catheter.

## Introduction

There is growing interest in the use of real-time magnetic resonance imaging-guided electrophysiology (MR-EP) to treat patients with cardiac arrhythmias.^1^^,^^2^ Potential advantages of MR-EP procedures include soft tissue visualization with a high contrast-to-noise ratio, improved assessment of arrhythmia structural substrate using late gadolinium enhancement (LGE) scar imaging, navigation of catheters using dedicated tracking techniques, online monitoring of ablation lesion formation and an evaluation of anatomic and physiologic changes during mapping and lesion delivery.^3^

Although most preliminary real-time MR-EP studies have been performed in the atria, where significant technical challenges remain for accurate substrate evaluation,^1^^,^^2^^,^^4^ magnetic resonance imaging (MRI) is the gold standard imaging modality for assessment of ventricular function and scar burden.^5^ Combined MR-EP techniques could offer synergistic benefits for the evaluation and ablation of ventricular tachycardia (VT) substrate. Previous studies using conventional systems and image integration where the association between electrical substrate for VT and MRI-derived scar have been investigated invariably report registration errors on a scale between 3.8 and 4.3 mm^6^^,^^7^ which could be a significant source of mismatch.^8^ Real-time MR-EP enables image registration to be performed within a single imaging modality, acquire imaging, and electrical data in the same co-ordinate system and minimize translational changes due to beat-to-beat cardiac motion and respiratory motion.

In this study, we describe the ability of a novel real-time MR-EP system to perform endocardial voltage mapping and limited assessments of delayed conduction in a porcine ischaemia–reperfusion model taking advantage of custom technical developments in a second generation MR-compatible catheter and a dedicated prototype image-guidance platform for interventional procedures. We hypothesized that with the minimization of registration errors and translational changes expected using a real-time MR-EP platform, an improved association between structural and electrophysiological substrate may be expected.

## Methods

### Animal model and infarct preparation

The research protocol was approved by the local institutional review board and complied with French law on animal experiments and the Guiding Principles for the Care and Use of Laboratory Animals published by the National Institutes of Health (8th Edition, National Academies Press, 2011). The research was performed at the Institut de Chirurgie Guidée par l’image (IHU), Strasbourg, France. Seven male domestic pigs (weight: 35.7 ± 5 kg; two healthy, five post-infarction) were treated with 800 mg amiodarone, twice daily for 4 days prior to and following an infarct procedure and/or imaging and electrophysiology studies. A closed-chest model of myocardial infarction was used as previously described^9^ (see Supplementary material online, *Figure S1* and *expanded methods*).

### Imaging study

All animals underwent a MRI scan for substrate assessment 6 weeks after infarct on a 1.5 T scanner (MAGNETOM, Aera, Siemens Healthcare, Erlangen, Germany). Each animal was sedated, intubated, and mechanically ventilated as per the infarct procedure for all imaging studies. A three-dimensional (3D) electrocardiogram (ECG)-triggered whole heart bSSFP MRI data set was acquired to enable manual segmentations of cardiac chambers (transverse slice orientation, anterior-posterior phase encoding, 256 × 256 in-plane matrix size, TR/TE/α = 3.7 ms/1.64 ms/90^°^, voxel size = 1.25 × 1.25 × 2.5 mm^3^, bandwidth = 895 Hx/Px, GRAPPA factor = 2). For scar imaging, contrast was administered (Gadovist, Bayer, Germany) at a dose of 0.2 mmol/kg. High-resolution 3D LGE imaging was performed using a free-breathing, respiratory navigator, and ECG-gated (in diastole) inversion recovery, b-SSFP sequence (TR/TE/α = 3.45 ms/1.5 ms/90°, field of view = 339 × 264 × 100 mm^3^, voxel size = 1.2 × 1.2 × 1.2 mm^3^, bandwidth = 895 Hz/Px, GRAPPA factor = 2, 2RR acquisition). The LGE sequence was run 10–15 min after administration of contrast. Based on the LGE-MRI, scar was manually segmented using a version of the Medical Imaging Interaction Toolkit (MITK, Heidelberg, Germany) with the full-width-half-maximum (FWHM) threshold used to define scar and help guide electroanatomic mapping (EAM) during the subsequent procedure.

### Interventional cardiovascular magnetic resonance image-guidance platform

**Figure 1:**
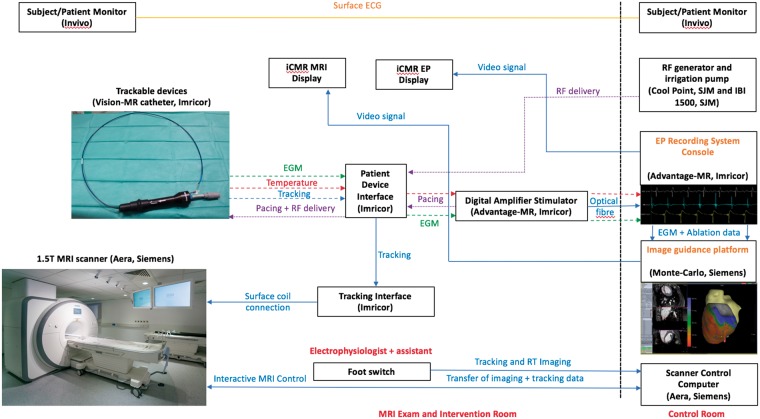
Set-up of the real-time MR-EP system to enable electrophysiology studies inside a MRI scanner. ECG, electrocardiogram; EGM, intra-cardiac electrogram; iCMR, interventional cardiovascular magnetic resonance; MR-EP, magnetic resonance imaging-guided electrophysiology; MRI, magnetic resonance imaging; RF, radiofrequency; RT, real-time.

**Figure 2:**
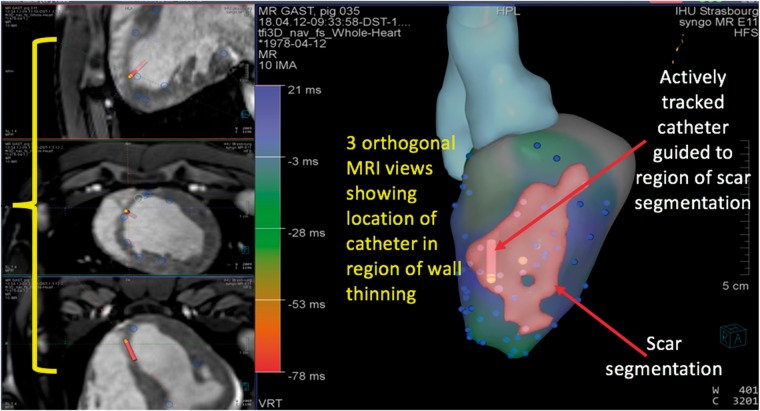
Representative depiction of image-guidance platform showing three orthogonal MRI views demonstrating location of catheter in relation to LV endocardium, three-dimensional segmentation of the left ventricle derived from MRI and scar segmentation from LGE images to guide EAM. EAM, electroanatomic mapping; LGE, late gadolinium enhancement; LV, left ventricular; MRI, magnetic resonance imaging.

A custom interventional cardiovascular magnetic resonance (iCMR) image-guidance platform (Siemens Healthcare, Erlangen, Germany) was used in this study (*Figures 1 and **2*). The application has the ability to load volumetric data from MRI scans, display multiplane reconstructions (MPRs) in three orthogonal planes and transfer segmentations of cardiac chambers derived from previous imaging or imaging acquired at the time of the EAM procedure. An automatic segmentation tool is incorporated within the software to ensure rapid image processing. During the MR-EP procedure, the MPR slices on the iCMR application can follow the tip of the actively tracked catheter to display 3D location of the catheter within the segmentations of the cardiac chambers as well as on the MPR images (*Figure 2*). The position of the actively tracked catheter is displayed following the implementation of a temporal smoothing algorithm that limits its excursion due to cardiac motion.

The software allows the imaging operator to start/stop sequences remote from the scanner console and configure parameters of each sequence on the MRI scanner. A MR-compatible foot-switch is also available as part of the application to start or pause an interactive imaging sequence that the electrophysiologist can operate (e.g. to use MPRs to navigate the catheter to a region of interest). The mapping interface of the application allows for changes to the rendering style or colour of a loaded segmentation, as well as place markers in regions of interest [e.g. to highlight intra-cardiac electrograms (EGMs) or mark sites of ablation]. The iCMR application communicates directly with the advantage EP recording system to display recorded activation times and voltage amplitudes. Colour interpolation is used to display this data, which is computed by a relaxation algorithm that takes the values on the mapping points as the fixed boundary condition and then performs a linear interpolation on the segmentation surface between these mapping points. These features enable the system to closely mimic that of a clinical EAM system whilst having additional capabilities to utilize imaging data for procedure guidance.

### Real-time magnetic resonance imaging-guided electrophysiology procedure

Vascular access was obtained via the femoral artery and vein under ultrasound guidance (9-Fr or 10-Fr introducer sheath) followed by administration of 100 units/kg of intravenous heparin. All EAM studies were performed inside the MRI scanner without the use of fluoroscopy at any point. The left ventricle (LV), right ventricle (RV), left atrium (LA), right atrium (RA), and aorta were manually segmented from the 3D ECG-triggered whole heart bSSFP MRI data set using the MITK-based platform. Image processing was performed during a 45-min window following the completion of imaging studies and prior to the start of EAM. During this time, each animal remained inside the scanner in order to minimize translational changes due to subject movement between imaging and mapping. The 3D shells of each chamber generated from the 3D whole-heart dataset, were imported into the iCMR guidance platform and used as a ‘road-map’ for mapping studies. The 3D segmentation of scar from the LGE-MRI was also imported into the iCMR guidance platform and overlaid onto the 3D shell for the LV chamber.

A custom 9 Fr, MR-compatible steerable catheter with a single gold 3.5-mm tip and ring bipolar electrode (3.5 mm inter-electrode spacing) and six circumferential open irrigation ports (Vision-MR, Imricor, Burnsville, MN, USA) was advanced into the LV cavity via retrograde aortic access. A number of modifications were implemented to the MR-compatible catheter from previous versions used in the atria^1^^,^^2^ to enable manipulation in the LV (Supplementary material online, *Figure S2*). These changes enabled improved torque transfer within the ventricle, manoeuvrability, and consistency of shape following deflection. The MR-compatible catheter has two solenoid micro-coils located 2 and 11 mm proximal to the ring electrode that enabled the location and orientation of the catheter to be detected in 3D space using a dedicated MRI active tracking sequence. A custom-built MR-EP recording system (Advantage-MR, Imricor, Burnsville, MN, USA) consisting of a digital amplifier, stimulator, and host workstation was used to record, display, and analyse intra-cardiac electrograms as previously described.^10^ A patient monitoring system suitable for use in the MRI environment (Invivo, Gainesville, FL, USA) was used to monitor a single lead ECG and invasive arterial blood pressure throughout the study.

### Active catheter tracking of magnetic resonance-compatible catheter

In order to accurately detect the location and orientation of the mapping catheter in 3D space, a dedicated active tracking sequence was used as described previously.^10^ Briefly, the X, Y, and Z co-ordinates of the catheter micro-coils were determined using the custom active tracking sequence, which was optionally interleaved with a fast balanced steady state free precession (bSSFP) imaging sequence automatically following the current catheter position. The active tracking sequence comprised three non-selective projection acquisitions along the respective axis. A dynamic imaging coil detuning approach and pre-spoiler were applied to avoid potential background noise, i.e. coil coupling and residual signal effects. Based on the acquired projections, the corresponding signal peaks were detected with a dynamic template-matching algorithm, which used the initial projections to calculate a template per coil and axis. The template was continuously updated with each new projection fulfilling a minimal peak-to-noise ratio to adapt to the changing shape of the projections while manoeuvring the catheter. The detected positions were fed back to both the iCMR platform (Siemens Healthcare) and the MRI scanner to update the rendered catheter position/orientation and imaging plane location, respectively.^10^

### Intra-cardiac electrogram recording and characterization

Activation and voltage data were acquired during sinus rhythm. For each sampling point, the local activation time (LAT) delay from a fixed intra-cardiac reference point to the initial deflection of the local LV electrogram was measured manually on the EP recording system and data transferred to the iCMR image-guidance platform. Similarly, the peak-to-peak voltage amplitude was also measured manually and transmitted to the guidance platform (*Figure 1*). Both data sets were used to generate colour-coded activation and voltage maps on the iCMR platform. Areas of focused mapping were based on the location of LGE-derived scar. In order to avoid EGM artefacts due to poor catheter-tissue contact, at least two consecutive EGMs had to have the same morphology prior to acceptance of each mapping point. Regions of abnormal myocardium were defined as areas with a bipolar voltage threshold <1.5 mV.^11^ EGMs were reviewed off-line at a sweep speed of 100 mm/s. After acquisition of activation and voltage maps, the LV catheter was used to pace during stable sinus rhythm (10 mA, 3 ms, cycle length 10% shorter than sinus cycle length) from sites of normal myocardium and scar. The time from the stimulus artefact to the surface QRS onset was used to distinguish regions of normal and delayed conduction. Following confirmation of capture, the time duration between the stimulus artefact and QRS onset was recorded. The MR-compatible catheter was sequentially manoeuvred to sites within normal myocardium and scar using active catheter tracking to generate a colour-coded map of stimulus-to-QRS (S-QRS) duration times. Sites with a S-QRS >40 ms during pace-mapping in sinus rhythm were considered regions of slow conduction as previously described.^12^ Following completion of the MR-EP procedure, pigs were euthanized with potassium chloride, and hearts were rapidly dissected for gross pathological examination. Hearts were photographed with areas of ischaemic scar delineated.

### Image registration, scar segmentation, and comparison to voltage maps

The LGE-MRI imaging was registered to the 3D whole heart MRI data sets using a point-based (landmark) rigid registration to guide EAM. Points were selected within the RV, LV, and LA blood pools of each image data set. Registration was performed on the Medical Imaging Interaction Toolkit (MITK; https://doi.org/10.1016/j.media.2005.04.005). Scar was segmented on the LGE-MRI using the FWHM method to normalize signal intensity relative to maximum myocardial signal intensity. First, the LV wall was manually segmented using a custom version of MITK. This was performed using the ‘Paint Tool’ on the MITK-based platform to derive the endocardial and epicardial border on a slice-by-slice basis with 3D interpolation to minimize discontinuities between slices. Then, the maximum signal intensity within the LV wall was computed and the voxels with signal intensity above 50% of the maximum intensity (FWHM) were labelled as scar.

To compare the scar segmentation with regions of low voltage, the scar segmentation was mapped onto the voltage map surface mesh. This was achieved in two steps. First, the scar segmentation image was rotated and translated so that it was aligned with the surface mesh. Second, the scar points were mapped onto the surface mesh using the iterative closest point method. In addition, the voltage map was converted to a binary map of scar (1) and normal tissue (0). In this ischaemia–reperfusion model, scar has been noted to be transmural in the majority of myocardial segments with LGE.^9^ The Sorensen–Dice similarity coefficient (DSC) between the two binary maps was then computed on a nodal basis for all regions, scar regions only and regions of normal myocardium. The DSC between LGE scar maps and voltage maps following thresholding at different cut-offs (0.5–3.5 mV) was also derived.

### Statistical analysis

Data analysis was performed using GraphPad Prism version 7.0 (GraphPad Software, CA, USA) or SPSS v24.0 (IBM Corp. Armonk, NY, USA). Continuous data are represented as mean ± standard deviation and compared using the Student’s two-tailed *t*-test. A two-sided *P* value <0.05 was considered statistically significant. For assessment of the accuracy of the MR-EP system to correctly identify scar and delayed conduction, the location of LGE-derived scar was taken as the ‘gold standard’ of structural substrate. The sensitivity, specificity, positive predictive value (PPV), and negative predictive value (NPV) of low-voltage points and S-QRS times using the MR-EP system to identify LGE-scar was assessed and used to derive receiver operator characteristic (ROC) curves.

## Results

All pigs that underwent a left anterior descending artery (LAD) infarct developed antero-septal scar which was visualized on the LGE images (mean scar volume: 6.80 ± 0.88 mL; Supplementary material online, *Figure S1*). There was no LGE present in healthy pigs that did not undergo the LAD infarct procedure.

### Real-time magnetic resonance imaging-guided electroanatomical mapping

**Figure 3:**
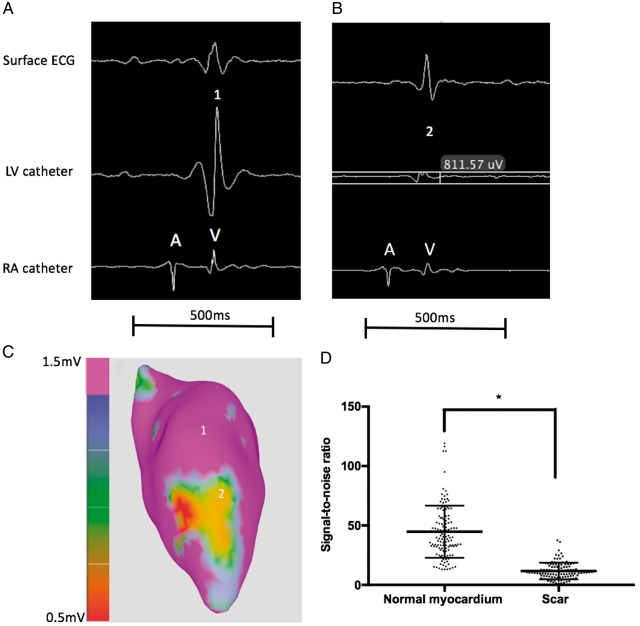
Representative examples of intra-cardiac EGMs obtained using the MR-EP system in a region of normal myocardium (Point 1) and area of scar (Point 2) (*A*–*C*). The baseline noise level inside the MRI scanner was in the region of 0.1 mV (∼10-fold higher than that in the conventional electrophysiology laboratory). Dot plot showing signal-to-noise ratios obtained for intra-cardiac EGMs in normal myocardium and LGE-derived scar regions from 7 animals; *P < 0.0001 (*D*). EGMs, intra-cardiac electrograms; LGE, late gadolinium enhancement; LV, left ventricular; MR-EP, magnetic resonance imaging-guided electrophysiology; MRI, magnetic resonance imaging; RA, right atrium.

**Figure 4:**
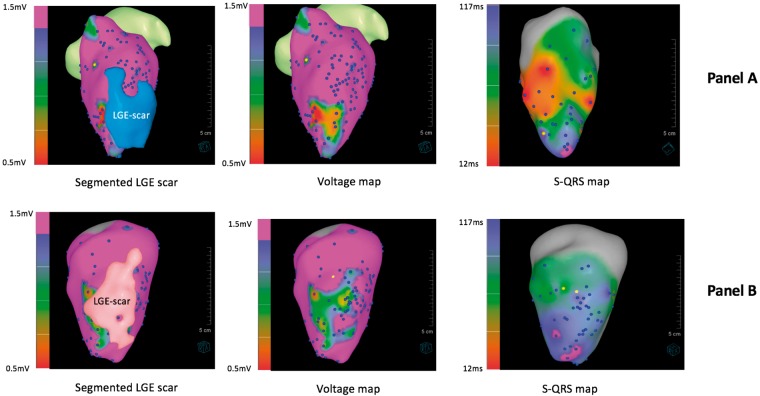
Representative examples of segmented LGE scar, voltage, and S-QRS maps obtained using real-time MR-EP system in two animals (*A* and *B*). LGE, late gadolinium enhancement; MR-EP, magnetic resonance imaging-guided electrophysiology; S-QRS, stimulus-to-QRS.

Segmentations of scar from the LGE-MRI were displayed on the iCMR image-guidance platform as coloured shells to guide EAM (*Figure 2*). 445 EGMs (range 30–186) were recorded from all animals in sinus rhythm (including 138 EGMs from regions located within the LGE scar segmentation). Using the MRI-derived LGE segmentation to differentiate between normal myocardium and scar, the mean signal-to-noise ratio of EGMs within normal tissue and scar was 44.78 ± 21.91 and 11.67 ± 6.99, respectively (*P* < 0.0001) (*Figure 3*). Pacing captured at 103 sites whilst 10 sites which were all in regions of LGE-derived scar did not capture; 56 (54.4%) sites had S-QRS delay ≤40 ms, 47 (45.6%) sites had a delay of ≥40 ms whilst 15 (14.5%) had a delay ≥80 ms. Representative examples of voltage and S-QRS maps obtained using the system are shown in *Figure 4*.

### Relationship between magnetic resonance imaging-derived scar, voltage, and delayed conduction

**Figure 5:**
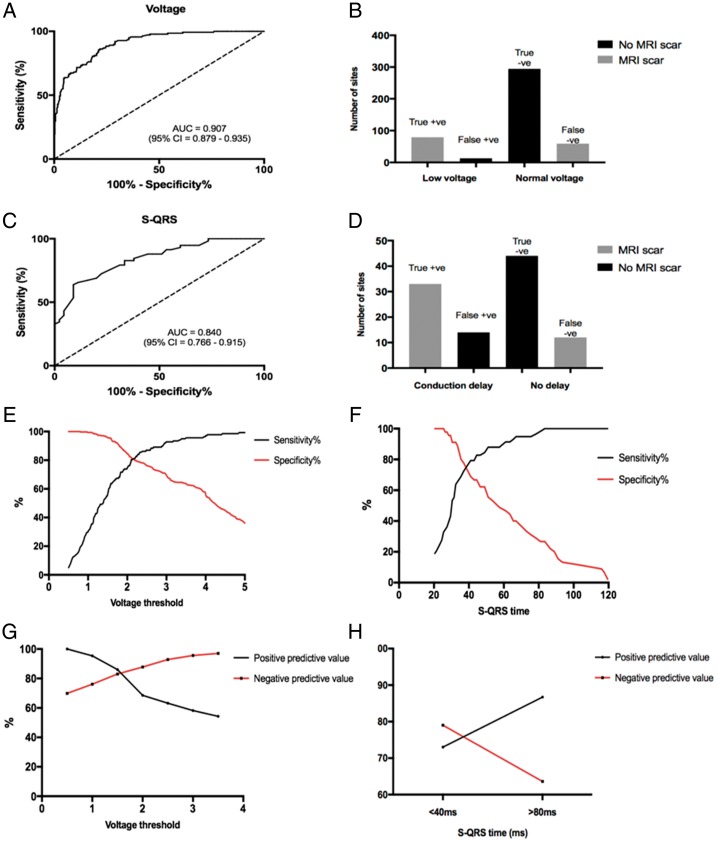
ROC curves (*A* and *C*) for prediction of LGE regions using voltage mapping and S-QRS. Frequency histograms (*B* and *D*) displaying the true positive, false positive, true negative, and false negative counts of voltage mapping and S-QRS measurements using the real-time MR-EP system to predict MRI-derived scar. Sensitivity, specificity, PPV, and NPV of measurements using the system using different normal voltage cut-offs and S-QRS times (*E–H*). LGE, late gadolinium enhancement; MR-EP, magnetic resonance imaging-guided electrophysiology; MRI, magnetic resonance imaging; NPV, negative predictive value; PPV, positive predictive value; ROC, receiver operator characteristic; S-QRS, stimulus-to-QRS.

Using conventional (0.5–1.5 mV) bipolar voltage thresholds, the sensitivity and specificity of voltage mapping using the MR-EP system to identify MR-derived LGE was 57% and 96%, respectively (ROC area under curve = 0.907; *P* < 0.0001). A S-QRS threshold of >40 ms using this system resulted in a sensitivity of 76% and specificity of 73% to identify MR-derived LGE (ROC area under curve = 0.840; *P* < 0.0001; *Figure 5*). At a threshold of 1.5 mV to define abnormal myocardium, the PPV and NPV of voltage mapping to identify LGE was 86% and 83%, respectively. At a threshold of 40 ms, the PPV and NPV of S-QRS time using the system to identify LGE was 73% and 79%, respectively (*Figure 5*).

**Figure 6:**
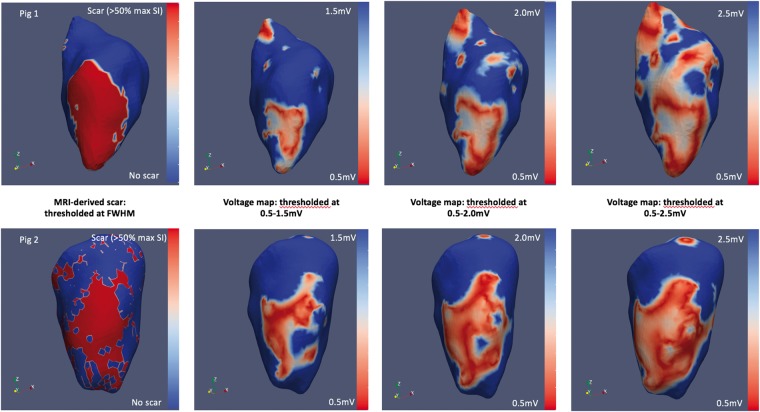
Sorenson–Dice similarity co-efficient between MR-derived scar shells (far left panels) and endocardial voltage maps with varying normal voltage thresholds (right panels) in two representative animals. FWHM, full-width-half-maximum; MR, magnetic resonance; MRI, magnetic resonance imaging.

**Figure 7:**
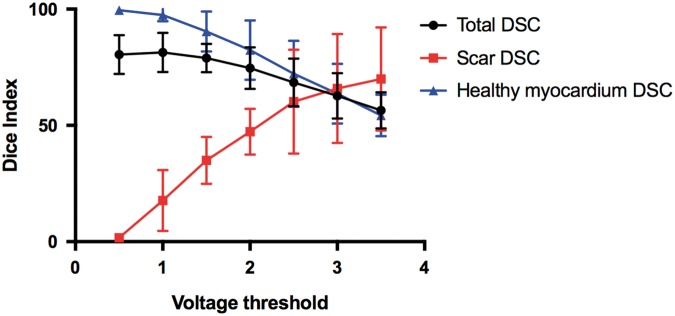
DSCs between MR-derived scar shells and endocardial voltage maps acquired using MR-EP system following application of normal cut-off thresholds of 0.5–3.5 mV. DSC is shown for overall similarity, similarity across scar nodes and normal myocardium nodes. DSC, Dice similarity co-efficients; MR, magnetic resonance; MR-EP, magnetic resonance imaging-guided electrophysiology.

There was a moderate relationship between low-voltage regions in the LV endocardium and LGE-derived scar mapped onto the endocardial surface mesh (*Figure 6*). At a voltage threshold of 1.5 mV, mean DSC across all nodes was 79.0% ± 6.0%, whilst mean DSC within scar regions only was 35.0% ± 10.1% and 90.4% ± 8.6% in normal myocardium regions only. An improvement in DSC within scar regions was observed using a higher voltage cut-off of 2.0 and 2.5 mV (47.3 ± 9.9% and 60.2 ± 22.4%) at the expense of reduced agreement across regions of normal myocardium (*Figure 7*).

## Discussion

This study shows that the prototype real-time MR-EP system can be used to guide catheters to regions of scar using active catheter tracking and to distinguish regions of low voltage and delayed conduction from healthy myocardium. There is a moderate relationship between low voltage and LGE scar using conventional bipolar voltage thresholds. An improved sensitivity for LGE detection may be achieved using higher bipolar voltage cut-offs with this system.

The relationship between local EGM amplitude and scar is complex, in part due to the dependence of voltage on infarct size, heterogeneity, and transmurality.^13^ Conventional bipolar voltage thresholds for scar detection may lack sensitivity to fully detect scar as variations in inter-electrode spacing and recording electrode size may affect the representation of EGMs^11^ Furthermore, although LGE-MRI is the current gold standard for visualization of ventricular scar post myocardial infarction, the limited spatial resolution of *in vivo* LGE-MRI can result in partial volume effects and limit the specificity of scar characterization.^13^ Increasing mapping resolution using multi-electrode catheters may also result in detection of a smaller area of low bipolar voltage as each data point represents a smaller tissue area with less far-field contamination.^14^ The use of multi-electrode catheters could improve the correlation between EAM and imaging as has been shown in a randomized study.^15^ An additional source of discrepancy when correlating EAM and pre-procedural imaging is registration error due to translational changes (patient movement, cardiac, or respiratory motion) or changes in volume, orientation, or rhythm of the heart between time of imaging and EAM.^16^

The real-time MR-EP system minimizes registration error through registration of electrical and structural data within a single imaging modality with the same co-ordinate system. The 3D whole heart sequence used for chamber segmentation was acquired during the same phase of the cardiac cycle as the 3D LGE to minimize translational changes due to beat-to-beat cardiac motion. Furthermore, both sequences were performed when animals were under general anaesthesia with reduced variability in respiratory motion, thereby minimizing translational changes due to respiratory motion. Compared to image integration approaches, where positional errors are introduced when registering catheter position to pre-procedural imaging, the MR-EP system tracks catheter position directly using a dedicated tracking sequence that is acquired in the same co-ordinate system as the 3D whole heart and LGE scans. The main sources of error with the MR-EP system include within scan registration error and catheter tip displacement on the 3D shell with the active tracking sequence. In a cohort of conscious patients scanned with an angiography sequence to create an endocardial mask and a 3D LGE acquisition, the within scan translation error was noted to be 1.9 ± 1.6 mm with a rotation error of 0.62 ± 0.41^°^.^17^ This is, however, likely to overestimate within scar error with the MR-EP system where translational movements were minimal as animals were under general anaesthesia. Using *ex vivo* technical validation, the average tip displacement of the actively tracked catheter using the MR-EP system was measured as 0.90 ± 0.58 mm along the axis of the catheter^1^ and is likely to be the best estimate of error with this set-up.

In this study, we show that despite the minimization of registration and translational errors, the relationship between scar delineated using a custom MR-compatible catheter and high-resolution isotropic LGE imaging (1.2 mm^3^) remains moderate when using standard voltage thresholds. An improvement in scar concordance with this system can be achieved using a higher normal bipolar voltage cut-off. Some investigators have found that abnormal potentials targeted for ablation may be present in tissue classified as ‘normal’ (>1.5 mV) voltage and manual adjustment of bipolar voltage thresholds to higher cut-off values may identify more confluent scar regions incorporating all abnormal signals.^18^ Regions of slow conduction could also be present in tissue of normal bipolar voltage and unmasked during extrastimulus pacing.^19^

Although the majority of real-time MR-EP studies published previously have focused on the atria, the full potential of substrate and lesion assessment afforded by such systems is likely to be realized in the context of VT ablation. There are limited data available evaluating real-time MR-EP systems in the ventricle.^20–22^ Our study builds on previous work to characterize the relationship between LGE-derived scar and electrophysiological measurements of low voltage and delayed conduction inside a MRI scanner.

Currently, limited visualization of soft tissue structures is possible in the electrophysiology laboratory with the use of intra-cardiac ultrasound (ICE), however, MRI offers an improved contrast-to-noise ratio and ability to acquire 3D whole heart images or 2D slices in any imaging plane. Furthermore, tissue characterization techniques such as LGE can be used to identify arrhythmogenic substrate whilst dedicated sequences can be used to monitor tissue temperature during ablation and provide a real-time method of calculating lethal thermal dose.^10^ The novel MR-EP system described is capable of visualizing the location and orientation of catheters relative to soft tissue, assess scar with MRI at the time of EAM, enable rapid segmentation and registration of cardiac chambers and potentially monitor formation of ablation lesions.^10^ These features of the MR-EP system could offer an alternative to conventional fluoroscopy-guided or ICE-guided procedures and improve catheter navigation, delivery of therapy, and assess anatomical and physiological changes during VT ablation with the potential to reduce risks and improve outcomes.

A number of technical developments are required prior to the realization of real-time MRI-guided VT ablation. The development of a MRI-compatible defibrillation system will be a prerequisite prior to any clinical studies and prototypes are currently under evaluation.^23^ Current surface ECG monitoring systems inside a MRI scanner are limited to 4–6 surface electrodes; in order to aid the diagnostic electrophysiology requirements of VT ablation, robust 12-lead ECG systems are required. Although high-fidelity 12-lead ECG recordings are possible,^24^ the impact of magneto-hydrodynamic effects and gradient switching-induced voltages within the MRI scanner can still corrupt ECG signals. There is currently a limited availability of MR-compatible devices; the development of MR-compatible multi-electrode catheters with similar capabilities to their conventional counterparts will accelerate progress in the electrophysiological assessment of substrate inside the scanner.^25^

### Limitations

There are several important limitations to this study. We did not define the bipolar voltage threshold that best correlates to histological scar using the MR-compatible catheter—rather two indirect methods of scar assessment were compared to each other. During assessment of S-QRS intervals to assess slow conduction, a single ECG lead was used to derive measurements due to the lack of availability of a MRI-compatible 12-lead ECG; as a result, no assessment of QRS morphology using a 12-lead ECG was performed during pacing. These measurements should, therefore, be interpreted with caution as we could not account for local latency although this would be expected to be minimal at the pacing cycle length used. Furthermore, the technique of S-QRS measurements may have limited sensitivity for the detection of regions of myocardium with slow conduction compared to an approach analysing the evoked response to extrastimuli.^19^ In this model, haemodynamic compromise and death of the animal was inevitable if VT was induced. As there was no means to defibrillate the animal inside the scanner, we deliberately avoided the induction of VT which in turn precluded activation and entrainment mapping. The MR-EP system used in this study consisted of a single electrode catheter and required manual annotation of activation times and voltages for each point on the EP recording system resulting in substantially lower mapping densities than with contemporary EAM systems. This could have lowered the precision of the sensitivity and specificity measures reported in the study. The development of automated mapping systems and multi-polar catheters for use inside the MRI scanner could better define the relationship between electrophysiological substrate and MR-derived substrate.

## Conclusions

There is a moderate association between low-voltage regions and sites of altered conduction determined using a novel real-time MR-EP system with scar derived from LGE-MRI. An improved sensitivity for LGE detection could be achieved using a higher normal voltage cut-off with this system and the respective catheter. Further technical developments in MR-compatible devices will accelerate progress towards real-time MRI-guided VT ablation.

## Supplementary material

Supplementary material is available at *Europace* online.

## Supplementary Material

euz165_Supplementary_Data
