# Supplementary material for: Evaluation of a real-time magnetic resonance imaging-guided electrophysiology system for structural and electrophysiological ventricular tachycardia substrate assessment
Source: Europace. 2019 Jun 20;21(9):1432–41. doi: 10.1093/europace/euz165 (PMC6735875; doi:10.1093/europace/euz165)
Supplement: euz165_Supplementary_Data [file euz165_supplementary_data.docx]

**Supplementary material**

**Mukherjee et al.**

**Expanded methods**

*Closed-chest model and infarct preparation*

Following pre-sedation with a combination of intramuscular tiletamine and zolazepam, endotracheal intubation was performed in domestic pigs and general anaesthesia induced and maintained with inhaled isoflurane (1.0 - 2.5%) with mechanical ventilation at 15-20 breaths/min. Percutaneous vascular access was gained from the right femoral artery (7-Fr introducer sheath) under ultrasound guidance. Following arterial access, a bolus of 7000 units of unfractionated heparin was administered intravenously followed by a maintenance infusion of 1000-2000 units/hr. An intravenous lidocaine infusion (1mg/min) was also initiated to reduce the risk of ventricular arrhythmias as previously described.^1^ A 6-Fr Hockey-stick guide catheter (Medtronic, Minneapolis, MN) was advanced to the left main stem and a 0.14in Choice PT extra-support angioplasty guidewire (Boston Scientific, Malborough, MA) was placed in the left anterior descending artery (LAD). A 3.0 x 12mm Emerge Monorail PTCA dilatation catheter (Boston Scientific, Malborough, MA) was placed over the angioplasty wire in the LAD. A coronary angiogram was performed to assess pre-infarct anatomy, following which the balloon was inflated to 12-14 atm in the mid-LAD distal to the second diagonal branch and maintained for 3 hours. A coronary angiogram was performed at 1 hour and 3 hours after inflation of the balloon to confirm that the artery was still occluded. The creation of infarct was also confirmed by the presence of ST-segment changes in lead V1. At the end of the procedure, 0.03mg/kg buprenorphine was administered intramuscularly and a fentanyl patch (125 micrograms/hour) was applied for 72 hours. Animals then underwent a 6-week recovery period prior to imaging and electrophysiology study.


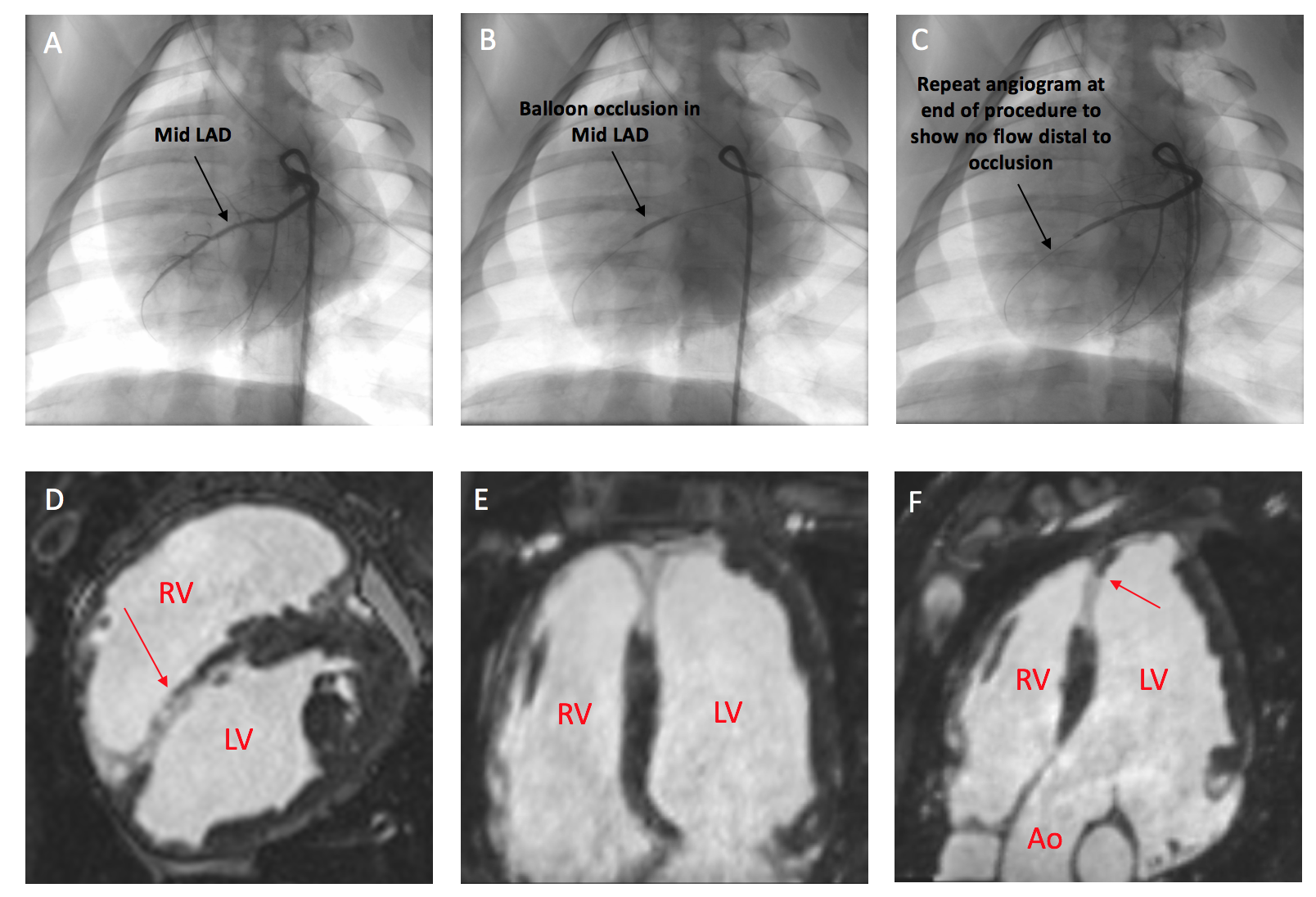


Supplementary Figure 1: Infarct creation in porcine model with mid-LAD balloon occlusion (A-C) with resulting LGE-MRI images acquired 6 weeks post infarct showing region of anteroseptal scar (D-E) - short-axis, 4-chamber and 3-chamber views shown. Red arrows show location and extent of LGE.

Supplementary Figure 2: Changes made in the 2nd generation MR-compatible Vision, Imricor ablation catheter to enable retrograde aortic access and improved manoeuvrability within the left ventricle. Previous clinical studies published with the Imricor catheter were in patients with atrial flutter using a 1st generation catheter. Images courtesy of Mr Tom Lloyd (Imricor Medical Systems).


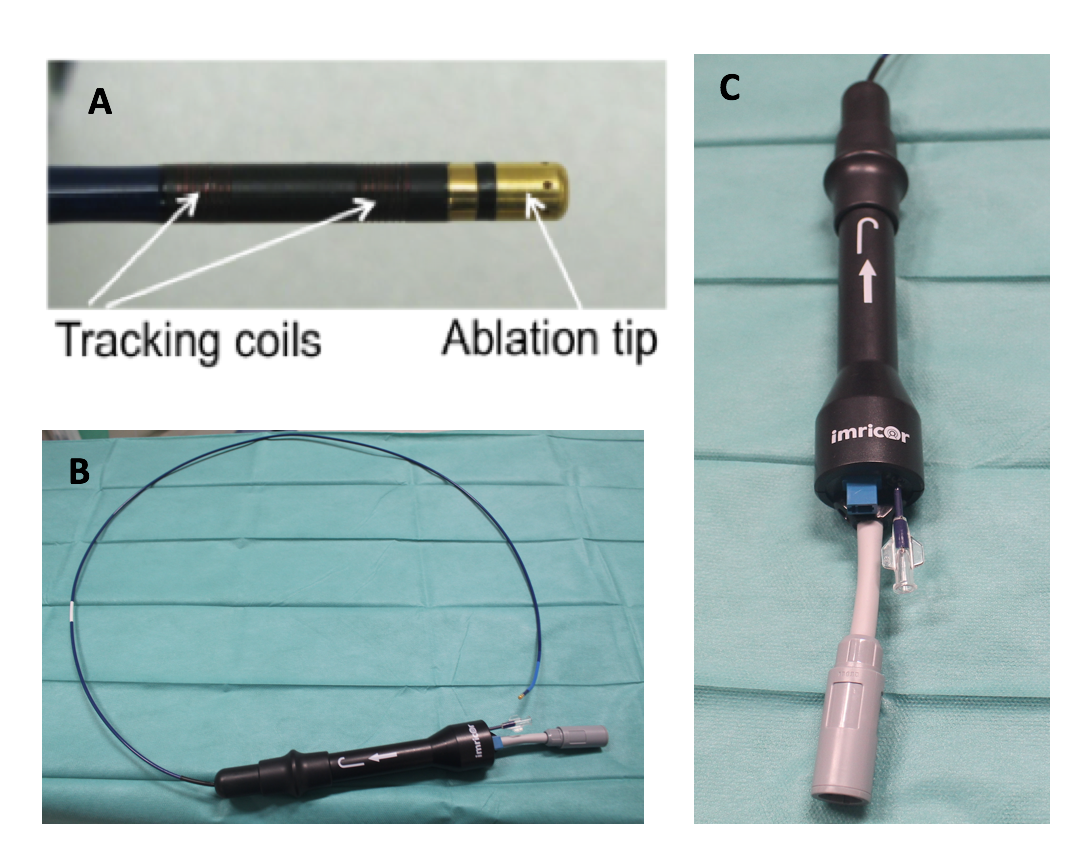


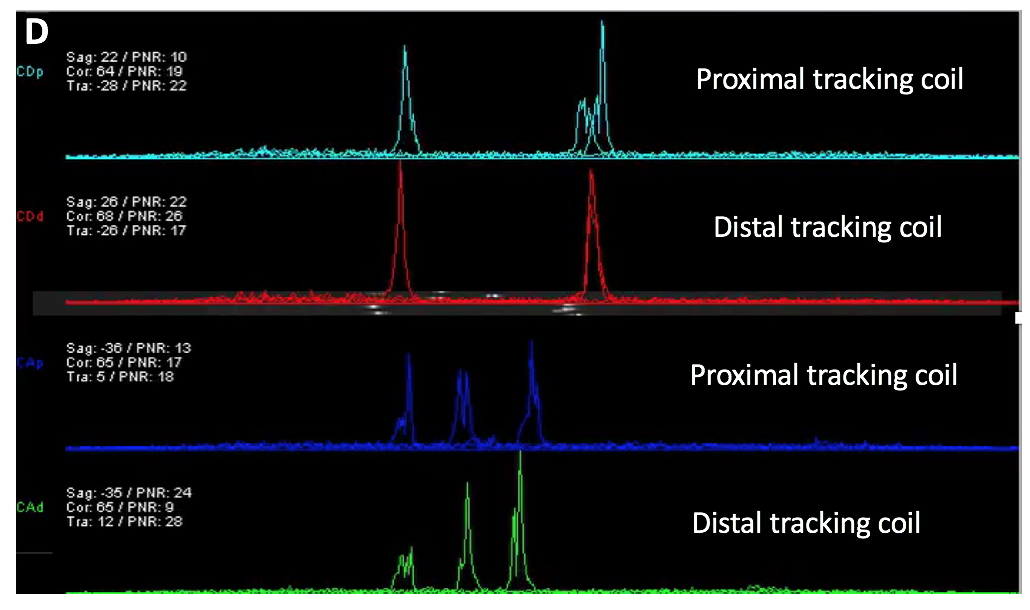


Supplementary Figure 3: Example images of catheter tracking coils (A) which are used to determine catheter position in 3D space using the active tracking sequence. The catheter and handle (B and C) have been optimised to operate within the ventricle inside a MRI scanner. The robustness of the active tracking sequence can be assessed from the signal-to-noise ratio of the proximal and distal tracking coils which are displayed (D) when the sequence is run on the MRI scanner. The image (D) shown represents the signal from the tracking coils of 2 separate catheters - one in the right atrium (top 2 signals) and one in the left ventricle (bottom 2 signals).


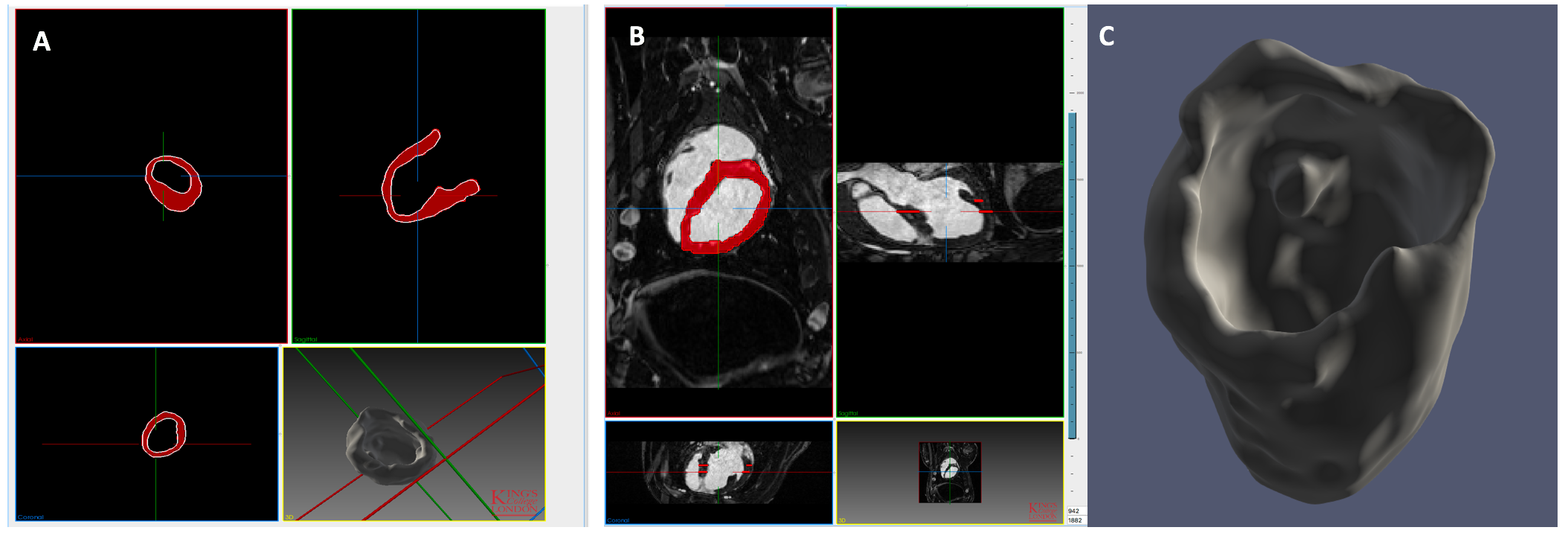


Supplementary Figure 4: Representative examples of segmentations of the LV wall (A and B) from 3D LGE-MRI images generated using the custom version of MITK and corresponding 3D shell (C) generated.


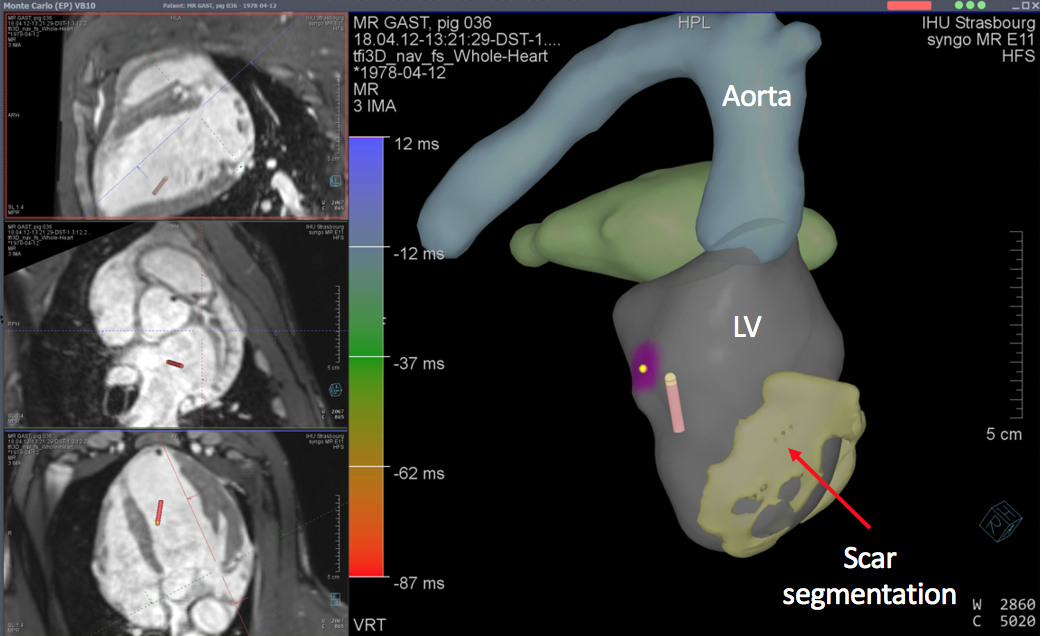


Supplementary Figure 5: The iCMR image guidance platform (Siemens Healthcare, Erlangen, Germany) can load volumetric data acquired from MRI scans (left) which are displayed as multi-planar reconstructions in 3 orthogonal views as well as load segmentations of cardiac chambers from the MRI data on the mapping interface (right). Acquired LAT and voltage points can then be displayed on the segmentations. These features allow the guidance platform the closely mimic that of a clinical-style EAM system whilst having custom capabilities to perform RT-MRI-guided EAM.

**Additional references:**

^1^ Tschabrunn CM, Roujol S, Nezafat R, Faulkner-Jones B, Buxton AE, Josephson ME, Anter E. A swine model of infarct-related re-entrant ventricular tachycardia: electroanatomic, magnetic resonance and histopathological characterization. Heart Rhythm 2016: 13; 262-73.

^2^ Mukherjee RK, Roujol S, Chubb H, Harrison J, Williams S, Whitaker J, O’Neill L, Silberbauer J, Neji R, Schneider R, Pohl T, Lloyd T, O’Neill M, Razavi R. Epicardial electroanatomical mapping, radiofrequency ablation and lesion imaging in the porcine left ventricle under real-time magnetic resonance imaging guidance - an in-vivo feasibility study. Europace 2017: Dec 26. Epub ahead of print.
